# The Effects of Cellulose on α-Amylase and α-Glucosidase Inhibition by *Aronia melanocarpa* Phenolic Compounds After Simulated Digestion

**DOI:** 10.3390/molecules31132250

**Published:** 2026-06-26

**Authors:** Lidija Jakobek, Ivica Strelec, Petra Matić

**Affiliations:** Faculty of Food Technology Osijek, Josip Juraj Strossmayer University of Osijek, Franje Kuhača 18, 31000 Osijek, Croatia; ivica.strelec@ptfos.hr (I.S.); petra.matic@ptfos.hr (P.M.)

**Keywords:** food matrix, diabetes, stomach, small intestine, aronia, enzyme inhibition, HPLC

## Abstract

The interaction with various food matrix constituents can affect the beneficial effects of phenolic compounds in the gastrointestinal tract. In this study, the effect of cellulose on the potential of phenolic compounds from *Aronia melanocarpa* after simulated gastrointestinal digestion to inhibit activity of enzymes important in carbohydrate digestion in vitro was investigated. Enzyme inhibitory activity was assessed by the inhibition of α-amylase and α-glucosidase. Cellulose was studied at three different levels. Phenolic compounds were released in the stomach (61–69%) and small intestine (58–66%). Cellulose increased recovery in the stomach (*p* < 0.05) and decreased it in the small intestine (*p* < 0.05), and the influence was dose-dependent. After digestion, phenolic compounds inhibited α-amylase and α-glucosidase. Cellulose increased the inhibition of α-amylase (*p* < 0.05), while it did not affect the inhibition of α-glucosidase. In conclusion, phenolic compounds after gastrointestinal digestion in the presence of cellulose can still inhibit α-amylase and α-glucosidase activity. Since the results represent only the in vitro model of digestion, further studies should focus on in vivo studies, and the effects of different food sources of cellulose to confirm the results.

## 1. Introduction

Diabetes affects people worldwide. Common risk factors for type 2 diabetes mellitus include lifestyle choices, genetic predisposition, and obesity. The costs of treating patients with diabetes are high and place a significant burden on health systems. Various medications, combined with lifestyle changes, are used to manage diabetes. However, medications cannot completely cure the disease. Furthermore, antidiabetic drugs may cause side effects such as nausea, diarrhea, or stomach pain. A proper diet containing active compounds beneficial for diabetic patients can help manage the consequences of diabetes and prevent its development [1].

Many studies have shown that phenolic compounds from plant-based foods have high potential for managing diabetes mellitus [2,3,4]. One way phenolic compounds can be beneficial is by mitigating hyperglycemia, or high blood glucose levels, especially after a meal [2,3,4]. After a meal, the breakdown of carbohydrates begins in the mouth, where salivary α-amylase breaks down polysaccharides into smaller disaccharides. This process continues in the small intestine with pancreatic α-amylase. Additionally, α-glucosidase located in the brush border of enterocytes in the small intestine further breaks down these molecules into glucose and other monosaccharides, which are then absorbed. Inhibiting these digestive enzymes may reduce glucose absorption and mitigate the rise in glucose levels after a meal, which can benefit patients with hyperglycemia [1].

Phenolic compounds from many plant sources can inhibit the activity of pancreatic α-amylase and α-glucosidase [2,3,4,5,6,7]. However, in the digestive tract, phenolic compounds can interact with other food matrix constituents (dietary fibers, proteins, other carbohydrates), which can influence enzyme inhibition. Cellulose is a component of the matrix in many plant-based foods. Since dietary fibers such as cellulose do not degrade in the stomach or small intestine, they can interact with phenolic compounds and affect their inhibition of enzymes.

Interactions between phenolic compounds and cellulose, including the binding of phenolic compounds to cellulose and the formation of complexes, as well as the influence of these interactions on the beneficial effects of phenolic compounds in the gastrointestinal tract, have not yet been sufficiently investigated. Studies have examined the adsorption of phenolic compounds onto cellulose [8]. That study investigated the adsorption of phenolic compounds such as catechin, caffeic acid, and ferulic acid onto cellulose [8]. Due to the variety of plant phenolic compounds and their structural differences, it is necessary to understand the behavior of other phenolic compounds around cellulose. Earlier research also explored the incorporation of various phenolic compounds into cellulose derivatives such as cellulose-based hydrogels [9], ethyl cellulose [10], and magnesium-grafted bacterial cellulose [11]. The aim of these studies was to create carriers from cellulose derivatives to deliver bonded phenolic compounds to the colon, where they can exert beneficial activities, or to use them in functional foods [10] and in dermatological and medicinal applications [9,11]. However, there is still a lack of knowledge about the interaction between native cellulose from food sources and phenolic compounds within the gastrointestinal tract, which would provide more information about the everyday diet when cellulose-containing foods are consumed together with foods that contain phenolic compounds. A better understanding of the interactions between cellulose and phenolic compounds within the digestive tract, and the influence of these interactions on enzyme inhibition, still requires further study.

Chokeberry, or *Aronia melanocarpa*, is a rich source of phenolic compounds [12,13,14]. It is known that phenolic compounds from chokeberries can inhibit the activity of α-amylase and α-glucosidase [4,7]. However, to the best of our knowledge, the effect of cellulose on the ability of phenolic compounds from chokeberries, after digestion, to inhibit α-amylase and α-glucosidase has not been explained.

In our previous work, we studied the interaction of *Aronia melanocarpa* phenolic compounds with dietary fibers such as β-glucan in model systems [13], after gastrointestinal digestion [14], as well as the influence of β-glucan on the beneficial effects of aronia polyphenols post-digestion, including potential antiradical activity and inhibition of α-amylase and α-glucosidase [15]. Examining the effects of different fibers such as cellulose on the beneficial effects of aronia polyphenols after digestion is a continuation of our research into the role of dietary fibers in digestion and the beneficial effects of polyphenols.

The aim of this study was to investigate the effect of cellulose on the inhibition of α-amylase and α-glucosidase by phenolic compounds from chokeberries after simulated gastrointestinal digestion. The digestion was conducted in vitro in the stomach and small intestine, both without cellulose (control sample) and with three different levels of cellulose. Phenolic compounds were determined before and after digestion using reversed-phase high-performance liquid chromatography (RP-HPLC). Enzyme inhibition was measured after digestion using spectrophotometric methods.

## 2. Results

### 2.1. Phenolic Compound Amount in the Digestion

**Figure 1:**
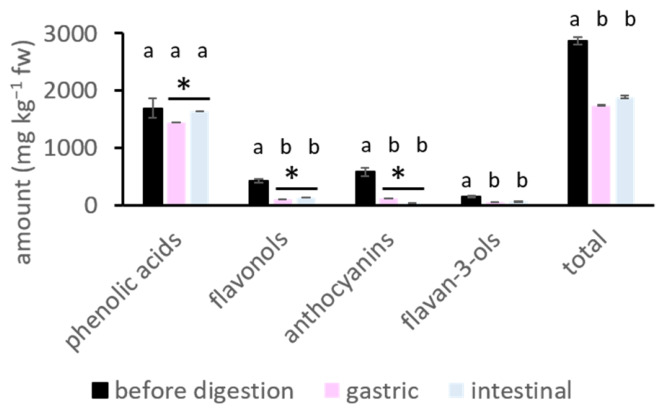
Amount of phenolic subgroups and total polyphenols (mg kg^−1^ fw) before digestion and after gastric and small intestinal phases. The extractions were performed in two parallel samples, each measured twice (*n* = 4). The digestion process was performed in five parallel samples, which were combined and analyzed twice (*n* = 2). Different letters indicate significantly different values according to the post hoc Tukey test (*p* < 0.05). Differences were also analyzed with a *t*-test, with * indicating a significant difference (*p* < 0.05). fw = fresh weight.

The main phenolic compounds identified in chokeberries belong to four subgroups: phenolic acids, flavonols, anthocyanins, and flavan-3-ols (Table 1), which is consistent with previously reported studies [13,14,15,16]. The amounts of individual and total phenolic compounds before and after digestion are shown in Table 1 and Figure 1, respectively. Phenolic compounds were released in the digestion. The amounts of flavonols, anthocyanins, flavan-3-ols, and total polyphenols significantly decreased in the stomach and small intestine compared to those in natural fruits (*p* < 0.05) (Figure 1).

The comparison of the amount present in two parts of the gastrointestinal tract, the stomach and small intestine, shows that the total amount was similar in both the stomach and small intestine (Figure 1). In contrast, earlier studies have shown either an increase [12] or a decrease [17] in the amount from the gastric to the small intestinal phase. However, the amounts of specific subgroups differed. The amount of anthocyanins decreased (*p* < 0.05) in the small intestine compared to the stomach. This decrease has already been reported and explained [16,17,18]. It might be due to the biotransformation of anthocyanins with changes in pH. At the low pH of the stomach, anthocyanins are in the flavylium cation form. At the higher pH of the small intestine (pH 7), they biotransform into colorless carbinol pseudobase, which causes their detected amount to decrease. Phenolic acids, on the other hand, increased (*p* < 0.05) from the gastric to the intestinal phase. Neochlorogenic and chlorogenic acids, the main phenolic acids in chokeberry fruit, can isomerize into cryptochlorogenic acid at the higher pH of the small intestine [19]. As reported, due to isomerization of chlorogenic acid between pH 5 and 5.5, cryptochlorogenic acid is formed. If the pH is between 6 and 9, the result of the isomerization is neochlorogenic and cryptochlorogenic acids [19]. The isomerization of chlorogenic, neochlorogenic, and cryptochlorogenic acids in simulated salivary, gastric, and intestinal fluids was examined in our earlier work as well [20]. All three acids were stable in the simulated gastric solution. Each of them isomerized into all three forms in simulated intestinal fluid at pH 7 [20]. According to these studies and our earlier work, it can be suggested that the formation of cryptochlorogenic acid can result from the isomerization of naturally present chlorogenic and neochlorogenic acids. Cryptochlorogenic acid was indeed identified in the small intestinal phase (Table 1). Moreover, the pKa values for chlorogenic and neochlorogenic acids are 3.44 and 3.46, respectively [21], which suggests that at pH 7 in the small intestine, these two phenolic acids might be in a dissociated form. Regardless of isomerization and dissociation, their stability could have led to the increased amount of total phenolic acids in the small intestine. The increase in phenolic acids is similar to what was reported in the study by Kim et al. (2020) [18].

Flavonols also increased (*p* < 0.05) from the gastric to the end of the intestinal phase. Their forms depend on pH values. According to the pKa values of quercetin (1.8, 6.4, 8.1, 9, 9.6, and 11.3) [22], quercetin might form a non-dissociated molecule at the lower pH of the stomach, while at the higher pH of the small intestine (pH 7), the dominant form could have dissociated OH groups [22]. Even with possible dissociation, flavonols showed resistance and a higher amount in the small intestine.

### 2.2. The Effect of Cellulose on the Release

**Figure 2:**
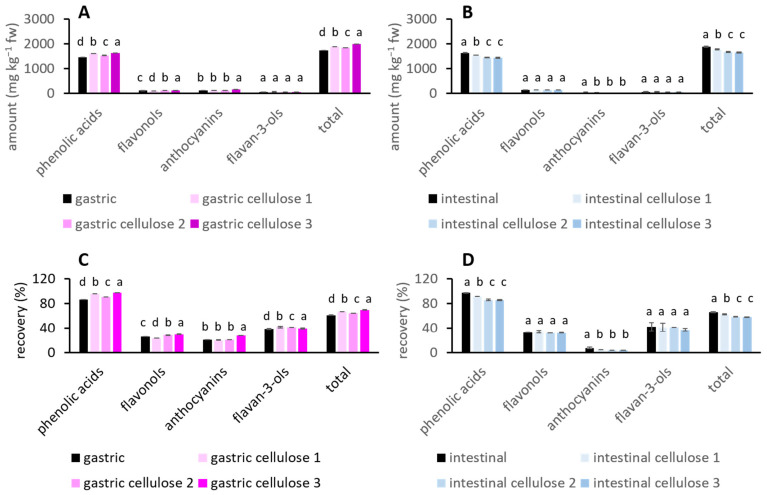
The influence of cellulose on the amount of phenolic compounds in (**A**) gastric and (**B**) intestinal digestion. The influence of cellulose on the recovery of phenolic compounds in (**C**) gastric and (**D**) intestinal digestion. The digestion process was performed in five parallel samples, which were combined and analyzed twice (*n* = 2). Different letters indicate significantly different values according to the post hoc Tukey test (*p* < 0.05). Digestion was conducted without and with added cellulose at three different levels (cellulose 1, cellulose 2, cellulose 3). fw = fresh weight.

Cellulose was added to the digestion of chokeberry at three different levels, from the lowest level 1 to the highest level 3. Figure 2A,B show the amounts of phenolic sub-groups and total phenolic compounds released in the stomach and small intestine.

With the addition of cellulose, the amounts of released phenolic compounds (phenolic acids, flavonols, anthocyanins, total phenolic compounds) increased in the stomach (*p* < 0.05) and decreased (phenolic acids, anthocyanins, total phenolic compounds) (*p* < 0.05) in the intestinal phase.

The recovery of phenolic compounds without or with cellulose is shown in Figure 2C,D. The recovery of total phenolic compounds ranged from 61% to 69% in the stomach and from 58% to 66% in the small intestine, depending on whether digestion occurred without or with added cellulose. These recoveries are similar to those reported in earlier studies [14,23]. The addition of cellulose from level 1 to 3 significantly increased the recovery of all phenolic groups and total phenolic compounds (*p* < 0.05) in the stomach, and decreased the recovery of phenolic acids, anthocyanins, and total phenolic compounds (*p* < 0.05) in the small intestine.

The released phenolic compounds in both phases of digestion were correlated with added cellulose to determine how phenolic compounds depend on cellulose levels. The correlation between the amount of phenolic compounds after gastric digestion and different levels of cellulose is strong and positive (except for flavan-3-ols) (Table S1), while the correlation between phenolic compounds after intestinal digestion and cellulose is moderate to strong and negative (Table S2). This suggests that the effect of cellulose may be dose dependent. With increased cellulose, phenolic compounds increase in the stomach and decrease in the intestine.

According to earlier studies, the presence of different food matrices can affect the release and recovery of chokeberry phenolic compounds by either decreasing or increasing it [23,24,25], depending on the food matrix. In the study by Köpsel et al. (2025) [23], chokeberry juice was mixed with different food matrices such as milk with 3.5% fat, milk with 1.5% fat, fat-free milk, soy milk, and oat milk. Oat and soy milk are rich in dietary fibers. Dietary fiber-rich oat and soy milk decreased the release of phenolic compounds at the end of digestion compared to pure chokeberry juice. This decrease is similar to the decrease observed after the small intestine in this study. In the study by Stanisavljević et al. (2015) [24], chokeberry juice was mixed with infant formula, which immediately decreased the amount of phenolic compounds, suggesting that the food matrix binds phenolic compounds. At the end of digestion, the amount increased, which is opposite to this study. In a study where aronia polyphenols were encapsulated in different carbohydrates and their mixtures [25], the recovery of phenolic compounds increased in the stomach, which is comparable to the increase observed in this study. However, encapsulation resulted in higher recovery of phenolic compounds after small intestinal digestion, which is opposite to our findings. These studies suggest that the food matrix [24] or dietary fibers [23] bind soluble phenolics from chokeberry, which can affect their amount during digestion.

The influence of the food matrix has also been studied for other sources of phenolic compounds, such as apples. When the release of phenolic compounds from the whole apple matrix and from apple extract without the matrix was investigated [26], the release of some phenolic groups increased at the end of digestion (flavonols, flavanols, hydroxybenzoic acids), while that of others decreased (hydroxycinnamic acids, dihydrochalcones). It was suggested that the food matrix affected these trends. The food matrix (cell wall material) protected some phenolic compounds against the digestive microenvironment, prolonged oxidation, and oxidizing and hydrolyzing agents. It might also have protected phenolics against components that could cause their precipitation, such as bile salts. In that case, the result was a higher amount of phenolic compounds in the presence of the food matrix [26].

According to the aforementioned studies [23,24,26] and the results of this study, it can be suggested that phenolic compounds interacted with cellulose, which protected them against the gastric phase microenvironment. The result might be a higher amount in the gastric phase. In the small intestine, cellulose might have interacted with phenolic compounds; however, the unfavorable microenvironment of higher pH eventually decreased their amount and recovery. It is known that phenolic compounds can interact with, and bind to cellulose [27,28]. Particular phenolic compounds such as cyanidin-3-glucoside, chlorogenic acid, ferulic acid, gallic acid, (+/−)-catechin [28], and procyanidin B2, phloridzin, and epicatechin [27] have been determined to adsorb onto cellulose, up to 0.6 g per g of cellulose [28]. Phenolic compounds of higher molecular weight bind at a higher level compared to those of lower molecular weight [27]. The binding is suggested to be non-covalent (hydrophobic interactions and H bonds) [27,28].

**Figure 3:**
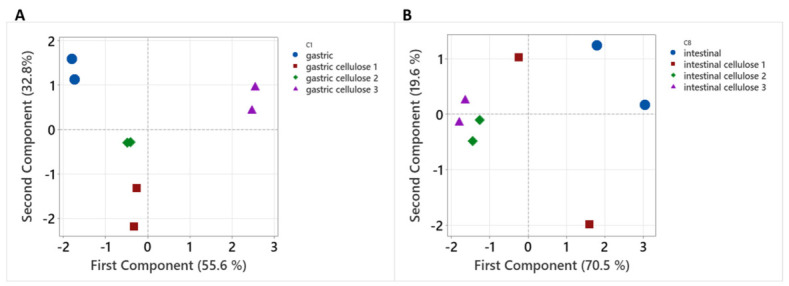
Principal component analysis (PCA) of the following data: amounts of total phenolic compounds and all phenolic subgroups (mg kg^−1^ fw) after (**A**) gastric and (**B**) intestinal digestion.

In the principal component analysis (PCA) (Figure 3), the amounts of phenolic compounds grouped according to digestion conducted without or with added cellulose, suggesting that the behavior of phenolic compounds in the presence or absence of cellulose is different.

Moreover, the increasing amounts of phenolic compounds in the gastric phase and the decreasing amounts in the intestinal phase of digestion when cellulose is present should probably not compromise their absorption, as suggested earlier (dos Santos Costa et al., 2015 [8]). However, this needs further study.

### 2.3. The Effect of Cellulose on the Inhibition of α-Amylase and α-Glucosidase

Phenolic compounds after small intestinal digestion inhibited α-amylase activity (*IC*_50_ 0.041 μmol of polyphenols) (Table 2). Earlier studies also reported that phenolic compounds from sweet and sour cherries [3], black mulberry [2], or chokeberry [4] inhibit α-amylase activity. In this study, the inhibition was significantly stronger (*p* < 0.05) when digestion was conducted with cellulose (*IC*_50_ 0.006, 0.026, and 0.015 μmol for cellulose levels 1, 2, and 3, respectively).

Cellulose itself is an inhibitor of α-amylase activity [29,30,31,32,33]. Cellulose was included in the digestion in this study. However, it was not present in the samples tested for inhibition after digestion. Samples were centrifuged and filtered after digestion to remove cellulose. To confirm this, the solubility of cellulose was studied in simulated digestion fluids (Table 3). It was shown that cellulose loss was small (2 to 3% in gastric and intestinal fluids), which suggests that cellulose is not soluble and was effectively removed from the liquid samples after digestion by centrifugation and filtration.

Additionally, digestive enzymes (α-amylase) are present in the samples after digestion. The influence of these enzymes, which originate from the digestion process and are present in samples tested for α-amylase inhibition, was controlled by conducting a blank digestion. In the blank digestion, water was used instead of chokeberry, and digestion was conducted without or with three levels of added cellulose. The samples after the blank digestion were used as controls in the α-amylase inhibition tests (as explained in Materials and Methods Section 4.7). By comparing the inhibition of α-amylase by chokeberry samples with that of control samples, the effects of enzymes originating from the digestion process were avoided.

Considering all of this, it can be suggested that released phenolic compounds do inhibit α-amylase. To see which phenolic subgroups might be important for the inhibition, the phenolic subgroups after intestinal digestion (μmol L^−1^) were correlated with α-amylase inhibition (*IC*_50_ in μmol). The correlation was not strong and it was visible that intestinal digestion conducted without cellulose differed from those with cellulose, and contributed to the low correlation (low R^2^ value). When the sample from digestion without cellulose was excluded, the correlation became strong (high R^2^ value) and negative (with the increasing amount of phenols, the *IC*_50_ is lower/stronger). Table 4 shows those correlations between the amounts of phenolic subgroups in intestinal digestion conducted with cellulose, and α-amylase inhibition. Phenolic acids, flavonols, and anthocyanins showed strong, and negative correlations with inhibition (R^2^ = 0.925, 0.796, 0.691, respectively), while the correlation was weaker for flavan-3-ols (R^2^ = 0.279). It is already shown that amongst phenolic compounds from chokeberries, phenolic acids (chlorogenic acid) and anthocyanins (cyanidin-3-glucoside) are the most effective α-amylase inhibitors [4], similar to this study. Flavonols (quercetin-3-glucoside and quercetin-3-glucoside and quercetin-3-galactoside) from chokeberry leaves showed strong correlation with the inhibition of α-amylase [7], similar to this study. Amongst several different phenolic compounds, chlorogenic acid inhibited α-amylase; however, its inhibition was weaker than that of epicatechin (flavan-3-ol) [34], opposite to this study.

The experiments on α-glucosidase inhibition were also compared to samples after blank digestion (control). However, blank digestion did not affect the results, probably because α-glucosidase is not present in the simulated digestion. Furthermore, cellulose was not present in the inhibition experiment. This suggests that the inhibition of α-glucosidase shown here represents the activity of released phenolic compounds. The activity of α-glucosidase was inhibited by phenolic compounds after small intestinal digestion (*IC*_50_ 0.020 μmol) (Table 2). Phenolic compounds from sweet and sour cherries [3] and black mulberry [2] also inhibited α-glucosidase activity, similar to this study. When cellulose was present in the digestion, the inhibition was similar (*IC*_50_ 0.016, 0.023, 0.017 μmol for cellulose levels 1, 2, and 3, respectively). It can be suggested that the presence of cellulose in the digestion did not affect the ability of the remaining phenolic compounds to inhibit α-glucosidase. In our earlier study, phenolic compounds inhibited α-glucosidase more strongly than acarbose (an antidiabetic drug) [15].

The inhibition was correlated with the amount of phenolic subgroups. Intestinal digestion without cellulose again differed from digestion with cellulose and contributed to the low correlation (low R^2^ value). When digestion without cellulose was excluded, the correlation improved (Table 4). Phenolic acids and flavonols showed moderate, negative correlations with inhibition (R^2^ = 0.477 and 0.484, respectively), while the correlation was negative and weak for anthocyanins and flavan-3-ols (R^2^ = 0.276 and 0.103). Flavonols from chokeberry leaves showed strong correlation with the α-glucosidase inhibitory activity [7] which agrees with this study. Anthocyanins from black mulberry were stronger α-glucosidase inhibitors than non-anthocyanin polyphenols [2], opposite to this study.

**Figure 4:**
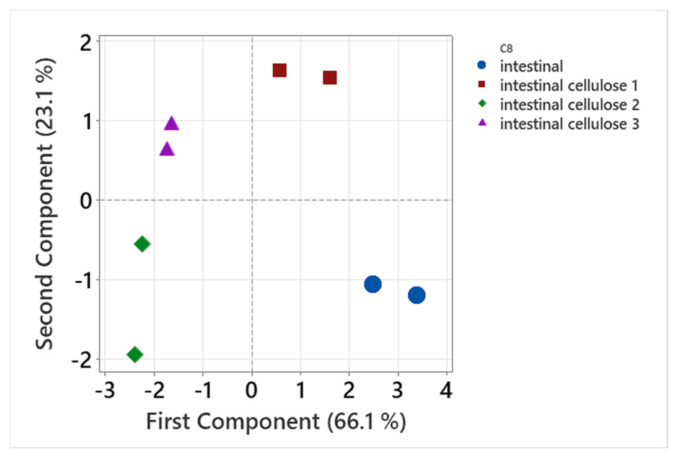
PCA plot of the following data: amount of cellulose (g); amounts of phenolic acids, flavonols, anthocyanins, flavan-3-ols, and total polyphenols after small intestinal digestion (mg); and *IC*_50_ for α-amylase and α-glucosidase (μmol).

PCA was used to further analyze the amounts of phenolic compounds after digestion, the amount of cellulose, and *IC*_50_ values (Figure 4).

The first component (66.1%) and the second component (23.1%) separated the data for intestinal digestion without cellulose and with different levels of cellulose. This suggests that the digestion and bioactivities of chokeberries without and with cellulose may be different.

## 3. Discussion

Elevated levels of cellulose increased both the amount and recovery of phenolic compounds from chokeberries in the stomach. These findings may result from the interaction with cellulose, which protected phenolic compounds from transformation at lower pH and provided greater stability. Phenolic compounds may have been adsorbed onto cellulose, forming phenolic compound–cellulose complexes that ultimately protected them from degradation. Similar results were reported in an earlier study [25]. An increased amount of phenolic compounds released in the stomach was observed for chokeberry phenolic compounds encapsulated with maltodextrin, maltodextrin plus carboxymethyl cellulose, gum Arabic, or xanthan gum, compared to the amount released when chokeberry polyphenols were not encapsulated [25]. A higher amount released in the stomach may be beneficial to health, as phenolic compounds can exert many positive effects in the stomach. One such effect is the mitigation of gastric ulcers, as shown for blueberry polyphenols [35] or the demonstration of reactive carbonyl trapping activity [36].

According to our study, elevated amounts of cellulose in the small intestine decreased both the amount and recovery of phenolic compounds. Lower recovery may result from the previously mentioned transformation of phenolic compounds due to the microenvironment of the small intestine, which decreases their amount, particularly anthocyanins. Additionally, lower recovery may result from the adsorption of phenolic compounds onto cellulose. Even though cellulose decreased the recovery of phenolic compounds, they may still have the potential for beneficial effects. Some of these effects were demonstrated in earlier studies [36,37]. If ingested with cellulose, chokeberry phenolics may still have the potential to provide beneficial effects, as they remain accessible in the small intestine.

Moreover, this study demonstrated the inhibition of α-amylase and α-glucosidase activity by phenolic compounds released from chokeberries in the small intestine, which may slow carbohydrate digestion in the gastrointestinal tract and be helpful in diabetes. The addition of cellulose increased the potential for α-amylase inhibition, while it did not appear to influence α-glucosidase inhibition. Earlier studies showed that cellulose itself inhibits α-amylase activity [29,30,32,33]. Cellulose binds to α-amylase through hydrogen bonding and Van der Waals forces [32] or hydrophobic forces [30,33], altering the secondary conformation and hydrophobicity of α-amylase [32], which ultimately inhibits its activity. However, in this study, cellulose was not present in the samples used for testing inhibition. It may be suggested that cellulose in our experiment did not affect the ability of released phenolic compounds to inhibit α-amylase and α-glucosidase.

However, this study is limited to the in vitro digestion model and includes only gastric and intestinal digestion, not the entire digestive process. In addition, coefficient of variation was higher for some detected compounds. Therefore, to confirm or expand our understanding of the influence of cellulose on enzyme inhibition, further research might involve in vivo studies. Moreover, this study used only pure cellulose as a model. Future studies should focus on using different foods commonly found in the diet that contain cellulose. Such studies may help in determining good dietary habits.

## 4. Materials and Methods

### 4.1. Chemicals and Solutions

Extrasynthese (Genay, France) was source for purchase of quercetin-3-galactoside, cyanidin-3-galactoside chloride, and cyanidin-3-glucoside chloride, and Sigma-Aldrich (St. Louis, MO, USA) to purchase neochlorogenic acid, (−)-epicatechin, quercetin-3-glucoside, quercetin-3-rutinoside, chlorogenic acid, and cryptochlorogenic acid. Some additional chemicals were also purchased from Sigma-Aldrich (St. Louis, MO, USA) such as α-amylase 13 U mg^−1^, pepsin 632 U mg^−1^, α-glucosidase 100 UN, pancreatin 8 USP, bile salt, 4-nitrophenyl α-D-glucopyranoside, and cellulose. Potassium sodium tartarate tetrahydrate, potassium dihydrogen phosphate, potassium chloride, calcium chloride, sodium hydrogen carbonate, magnesium chloride, and sodium dihydrogen phosphate dihydrate were obtained from Gram mol d.o.o. (Zagreb, Croatia), and ammonium carbonate from Kemika (Zagreb, Croatia). Sodium chloride, and sodium phosphate dibasic dodecahydrate were bought from Carlo Erba Reagents (Val de Reuil, France), methanol from J.T. Baker (Gliwice, Poland), ortho-phosphoric acid from Fluka (Buchs, Switzerland), and 3,5-dinitrosalicylic acid from Thermo Fisher Scientific (Waltham, MA, USA).

Stock solutions of electrolytes were prepared: 0.5 mol L^−1^ (KCl, KH_2_PO_4_, (NH_4_)_2_CO_3_), 1 mol L^−1^ (NaHCO_3_), 2 mol L^−1^ (NaCl), and 0.15 mol L^−1^ (MgCl_2_). Solutions for simulated digestion were prepared by diluting these stock solutions. Concentrations were in mmol L^−1^ as follows: simulated salivary fluid electrolyte solution SSF—18.9 KCl, 4.6 KH_2_PO_4_, 17 NaHCO_3_, 0.056 MgCl_2_, and 0.06 (NH_4_)_2_CO_3_; simulated gastric fluid electrolyte solution SGF—8.6 KCl, 1.1 KH_2_PO_4_, 31.3 NaHCO_3_, 0.15 MgCl_2_, 0.63 (NH_4_)_2_CO_3_, and 59 NaCl, the pH of SGF was then adjusted to 3 with 1 mol L^−1^ HCl; simulated intestinal fluid electrolyte solution SIF—8.5 KCl, 1 KH_2_PO_4_, 106.3 NaHCO_3_, 0.4 MgCl_2_, and 48 NaCl, the pH of SIF was adjusted to 7 with 1 mol L^−1^ HCl. The α-amylase (1500 U mL^−1^) was prepared in SSF, pepsin (20,000 U mL^−1^) in SGF, pancreatin (800 U mL^−1^) in SIF, and bile salts in SIF (25,000 mg L^−1^).

A color reagent used for α-amylase inhibition was prepared by mixing warm water (30 mL), potassium sodium tartarate tetrahydrate (20 mL, 5.3 mol L^−1^ in 2 mol L^−1^ NaOH), and 3,5-dinitrosalicylic acid (50 mL, 96 mmol L^−1^ in water). α-Amylase (2 U mL^−1^) was prepared in phosphate buffer (pH 6.9, 0.02 mol L^−1^), and starch (1%) in water. For α-glucosidase inhibition, solutions were prepared as follows: α-glucosidase (4 U mL^−1^ in buffer, pH 6.9, 0.1 mol L^−1^), and substrate 4-nitrophenyl α-D-glucopyranoside (pNGP) (5 mmol L^−1^ in buffer, pH 6.9, 0.1 mol L^−1^).

### 4.2. Sample Preparation

Chokeberry fruit was harvested in an orchard (Orahovica, Croatia). Known amount of chokeberries (100 g) was dried in a food dehydrator (KYS-328A, Delimano, Foshan City, China). Dried chokeberries were weighed (36.94 g) and ground in a coffee grinder. Dried samples were sealed in vacuum bags and frozen. The ratio of fresh weight (fw)/dried weight (dw) (100/36.94) was 2.707.

### 4.3. Extraction of Phenolic Compounds

Phenolic compounds were extracted from dried chokeberries in two steps. In the first step, 0.08 g of chokeberry was weighed into a plastic tube. Then, 1.5 mL of 80% methanol was added to the tube. The tube was placed in an ultrasonic bath (RK-100, Bandelin electronic GmbH, Berlin, Germany) for 30 min, centrifuged (Eppendorf Minispin, Eppendorf, Hamburg, Germany) for 5 min at 10,000 rpm, and the supernatant was separated from the residue. Next, 0.5 mL of 80% methanol was added to the residue in the tube and the extraction was repeated, followed by five additional extractions with 0.55 mL of 0.1% HCl in methanol. All supernatants were combined into one extract, filtered (0.2 μm PTFE), and analyzed using an RP-HPLC method.

In the second step, the residue was extracted with enzymes. In the plastic tube containing the residue from the previous extraction, 1.05 mL of distilled water, 60 μL of bile salts, 30 μL of pancreatin, and 15 μL of pepsin were added. The extraction was conducted in a dry block thermostat (Bio TDB-100, Biosan, Riga, Latvia) for 2 h at 37 °C. The tube was transferred to an ice bath, and then centrifuged for 5 min at 10,000 rpm. The supernatant was separated from the residue, filtered (0.2 μm PTFE), and analyzed with RP-HPLC.

After obtaining the concentration in mg L^−1^, the amount was recalculated as mg kg^−1^ of dry weight and then as mg kg^−1^ of fresh weight (fw) (mg of polyphenols/kg dry weight = mg of polyphenols/2.707 kg of fresh weight). Extractions were performed in duplicate and analyzed twice (*n* = 4). The amount of phenolic compounds before digestion was obtained by adding the amounts from the two extraction steps.

### 4.4. Simulated Digestion

Dry chokeberry sample was weighed (0.06 g) in 5 plastic tubes to which 175 μL of SSF, 49 μL of H_2_O, 1.3 μL of CaCl_2_, and 25 μL of α-amylase were added (salivary digestion). After 2 min in the dry block thermostat (37 °C), 375 μL of SGF, 15 μL of H_2_O, 0.3 μL of CaCl_2_, 10 μL of HCl (1 mol L^−1^), and 100 μL of pepsin were added in tubes to simulate gastric digestion. After 2 h (37 °C), tubes were placed in an ice bath, and then in the centrifuge (5 min, 10,000 rpm). Supernatants were separated from residues, and combined in one sample after the gastric digestion. A liquid sample of the gastric digestion was filtered (0.2 μm PTFE), and analyzed with RP-HPLC two times (*n* = 2). The amount in mg kg^−1^ dw was recalculated to mg kg^−1^ fw, as described in Section 4.3.

The sample of small intestine digestion was obtained by the same protocol after which small intestinal digestion continued by adding 550 μL of SIF, 180 μL of H_2_O, 2 μL of CaCl_2_ (0.3 mol L^−1^), 7.5 μL of NaOH (1 mol L^−1^), 250 μL of pancreatin, and 10 μL of bile salt. After 2 h in the dry-block thermostat (37 °C), samples were cooled in an ice bath, and centrifuged (5 min, 10,000 rpm). Supernatants were separated from residues, and combined in one sample after the small intestinal digestion, filtered (0.2 μm nylon), and used for the experiment of the enzyme inhibition. An aliquot was filtered (0.2 μm PTFE), and analyzed with RP-HPLC two times (*n* = 2). The amount in mg kg^−1^ dw was recalculated to mg kg^−1^ fw, as described in Section 4.3.

Simulated digestion with the addition of three levels of cellulose was conducted with the same procedure. For the cellulose level 1, each of five tubes contained chokeberry sample and cellulose (0.002 g). After the digestion the supernatants were combined to form the first sample, a cellulose level 1, with the final amount of cellulose 0.01 g. In the second (each of five tubes contained chokeberry and 0.004 g of cellulose) and third sample (each of five tubes contained chokeberry and 0.006 g of cellulose), the final amounts of cellulose were 0.02 and 0.03 g (cellulose level 2 and 3, respectively).

The recovery was calculated as:

(1) $$\left(recovery\right)\left(\%\right)=\frac{\gamma_{digestion\;phase}(mg\;{kg}^{-1})}{\gamma_{before\;digestion}(mg\;{kg}^{-1})}\times 100$$

*γ*_digestion phase_ represents mg of phenolic compound per kg of fresh weight fruit after the gastric or intestinal digestion phase, and *γ*_before digestion_ represents mg of phenolic compound per kg of fresh weight fruit before digestion.

Blank simulated digestion was conducted (blank for gastric digestion, blank for small intestinal digestion, blank for gastric digestion with three levels of added cellulose, and blank for intestinal digestion with three levels of added cellulose). The procedure was the same. The tubes contained water instead of chokeberry and the same amounts of cellulose as in the digestion.

### 4.5. Solubility of Cellulose in Simulated Fluids

Cellulose (0.02 g, *m*_0_) was weighed into three plastic tubes (*m*_plastic tube_), and 2 mL of simulated fluids (SSF, SGF, or SIF) were added to tubes, respectively. The solutions were placed on a shaker (IKA K 130, IKA-Werke, Staufen, Germany) for 24 h, then centrifuged for 5 min, and the simulated fluids were removed. The tubes, together with the wet residues, were weighed (*m*_tube+wet cellulose_), and the mass of wet cellulose was calculated as *m*_1_ = *m*_tube+wet cellulose_ − *m*_plastic tube_. The cellulose residues in the tubes were dried in an incubator (80 °C, 24 h). The tubes were then weighed (*m*_tube+dry cellulose_), and the mass of dry cellulose was calculated as *m*_2_ = *m*_tube+dry cellulose_ − *m*_plastic tube_. Experiment was done in two parallels (*n* = 2). The swelling degree (*SD*) and solubility (*S*) were calculated as follows:

(2) $$SD\left(\%\right)=\frac{m_{1}-m_{0}}{m_{0}}\times 100$$

(3) $$S\left(\%\right)=\frac{m_{0}-m_{2}}{m_{0}}\times 100$$

### 4.6. Reversed-Phase High-Performance Liquid Chromatography (RP-HPLC)

The HPLC Infinity II system included a quaternary pump, a PDA detector, and a vial sampler (Agilent Technologies, Santa Clara, CA, USA). Compounds were separated using a Poroshell 120 EC-C18 column (4.6 × 100 mm, 2.7 μm), and a Poroshell 120 EC-C18 4.6 mm guard column (Agilent Technologies, Santa Clara, CA, USA). The mobile phases were 0.5% H_3_PO_4_ in water (mobile phase A) and acetonitrile (mobile phase B). The gradient for increasing phase B was as follows: 5% at 0 min, 5 to 11% from 0 to 5 min, 11 to 15% from 5 to 7.5 min, 15 to 17.5% from 7.5 to 17.5 min, 17.5 to 20% from 17.5 to 20 min, 20 to 30% from 20 to 30 min, 30 to 70% from 30 to 32 min, 70% at 34 min, and 5% at 36 and 38 min. Linear calibration curves for phenolic compounds (r^2^ = 0.9942 − 0.9998) were used for quantification, while compounds were identification based on their retention times and UV/Vis spectra. Some compounds were tentatively identified (cyanidin-3-arabinoside and cyanidin-3-xyloside). Precision was expressed as the coefficient of variation (0 to 26.6%).

### 4.7. Inhibition of α-Amylase

Samples after intestinal digestion were examined as inhibitors of α-amylase. The reaction was carried out in plastic tubes with a total volume of 500 μL. The tubes contained varying volumes of phosphate buffer (pH 6.9, 0.02 M), 5, 10, 20, 40, or 60 μL of post-digestion sample, 240 μL of starch, and 150 μL of α-amylase. Briefly, phosphate buffer, starch, and the post-digestion sample were pipetted into the tubes, which were then placed in an incubator (IN 30, Memmert, Schwabach, Germany) at 37 °C for 10 min. After adding α-amylase, the tubes were incubated again at 37 °C for 10 min. Next, 250 μL of color reagent was added, and the tubes were placed in a water bath (LSB Aqua Pro, Grant Instruments, Cambridge, UK) at 100 °C for 10 min, then cooled. Finally, 2250 μL of water was added to dilute the mixtures. The absorbance (*A*_sample_) was measured at 540 nm using a UV/Vis spectrophotometer (UV 1280, Shimadzu, Kyoto, Japan). Tubes for the blank inhibition contained the same reagents except for the enzyme (*A*_sample blank_).

Enzymes present in samples after digestion can affect α-amylase inhibition in this experiment. To prevent this, samples from the blank digestion were used in a control experiment. For this control, the tubes contained all reagents as described for the inhibition assay, but samples after blank digestion (*A*_control_) were used instead of samples after digestion. Additionally, a control blank was prepared without enzyme (*A*_control blank_). The influence of enzymes present in the digestion on α-amylase inhibition was eliminated by comparing the sample inhibition (*A*_sample_ − *A*_sample blank_) with the inhibition of the blank digestion (*A_control_* − *A*_control blank_) as follows:

(4) $$\%\;inhibition=\frac{\left(A_{control}-A_{control\;blank}\right)-\left(A_{sample}-A_{sample\;blank}\right)}{\left(A_{control}-A_{control\;blank}\right)}\times 100$$

Concentrations of phenolic compounds in reaction mixtures (mg L^−1^) were recalculated in mol L^−1^ using the known molecular weights of the identified compounds. Based on the known volumes of post-digestion samples and the total volumes of the reaction mixtures, the concentrations of phenolic compounds were ultimately expressed in μmol. A diagram of percent inhibition versus phenolic compound concentration (μmol) was used to calculate the concentration of phenolic compounds required to inhibit 50% of the enzyme activity (*IC*_50_ in μmol). The inhibition assay was performed twice (*n* = 2).

### 4.8. Inhibition of α-Glucosidase

A total volume of 3000 μL was prepared in plastic tubes. These tubes contained varying volumes of sample after digestion (10, 20, 30, 40, 50, or 60 μL), phosphate buffer (pH 6.9, 0.1 M), p-NGP as substrate (250 μL), and α-glucosidase (250 μL). Buffer, p-NGP, and the post-digestion sample were pipetted into the tubes, which were then placed in the incubator (37 °C, 10 min). α-Glucosidase was added, and the tubes were incubated again (37 °C, 17 min). Absorbance (*A*_sample_) was measured at 405 nm. For the inhibition blank, tubes contained the same reagents except for the enzyme (*A*_sample blank_). In the control experiment, tubes contained the enzyme but no inhibitor (*A*_control_), while the control blank contained neither inhibitor nor enzyme (*A*_control blank_). The percent inhibition was calculated as shown in Equation 4. The concentrations of phenolic compounds in reaction mixtures were calculated in μmol, as explained in Section 4.7. The plot of % inhibition versus phenolic compound concentration (μmol) was used to calculate *IC*_50_ in μmol. The inhibition assay was performed twice (*n* = 2).

### 4.9. Statistical Analysis

The chemical and enzyme-assisted extractions were performed in two parallel samples, each measured twice (*n* = 4). The digestion process was performed in five parallel samples, which were combined and analyzed twice (*n* = 2). Post hoc Tukey test, *t*-test, principal component analysis (PCA), and Pearson coefficients were used to analyze the data. The software used for those analyses included Minitab 19 (Minitab LLC, State College, PA, USA), Statistica, version 14.2.0.18. (Cloud Software Group, Inc., Fort Lauderdale, FL, USA), and Microsoft Excel 2019 (Redmond, WA, USA). All independent measurements were analyzed with PCA.

## 5. Conclusions

This study investigated how a component of the food matrix, such as cellulose affects the ability of phenolic compounds from chokeberries to inhibit α-amylase and α-glucosidase after simulated digestion. Under in vitro conditions, cellulose increased the amount and recovery of phenolic compounds in the gastric phase and decreased the amount and recovery in the small intestinal phase. This effect was dose-dependent. After digestion in the small intestine, phenolic compounds retained their ability to inhibit the activity of α-amylase and α-glucosidase. In the presence of cellulose, phenolic compounds were still able to inhibit both enzymes. The results of this study could serve as a starting point for future research on the effects of foods rich in cellulose on enzyme inhibition. Such studies could provide a more realistic understanding of the effects of cellulose on inhibition.

## Figures and Tables

**Table 1:** The amount of phenolic compounds in chokeberry before and after gastric and small intestinal digestion (mg kg^−1^ fw). The digestion process was conducted without cellulose and with added cellulose at three different levels (level 1, level 2, level 3).

		Gastric				Intestinal			
			Gastric with Cellulose				Intestinal with Cellulose		
	Before	Gastric	Level 1	Level 2	Level 3	Intestinal	Level 1	Level 2	Level 3
Phenolic acids									
neochlorogenic acid	939.0 ± 90.9 ^a^	903.1 ± 0.5 ^ac^	1025.3 ± 0.0 ^a^	978.5 ± 0.1 ^a^	1025.7 ± 0.6 ^a^	767.0 ± 4.3 ^bc^	722.5 ± 4.4 ^b^	674.0 ± 11.7 ^b^	655.0 ± 6.5 ^b^
chlorogenic acid	756.4 ± 80.2 ^a^	549.5 ± 0.3 ^ab^	589.3 ± 0.9 ^ab^	556.2 ± 2.5 ^ab^	615.3 ± 0.7 ^b^	544.7 ± 4.7 ^ab^	484.8 ± 5.1 ^ab^	448.3 ± 5.9 ^c^	466.1 ± 9.4 ^ab^
cryptochlorogenic acid	n.d.	n.d.	n.d.	n.d.	n.d.	328.8 ± 3.3 ^ab^	342.1 ± 12.1 ^a^	328.3 ± 1.7 ^ab^	321.2 ± 1.5 ^b^
Flavonols									
unknown 2	75.3 ± 4.5 ^a^	20.2 ± 0.3 ^d^	19.7 ± 0.1 ^d^	22.3 ± 1.2 ^bd^	22.7 ± 0.0 ^bd^	30.4 ± 0.3 ^bc^	32.2 ± 2.4 ^c^	32.6 ± 0.1 ^c^	32.2 ± 0.1 ^c^
unknown 3	55.6 ± 1.5 ^a^	12.5 ± 0.1 ^c^	13.5 ± 0.1 ^c^	14.9 ± 0.6 ^c^	13.9 ± 0.2 ^c^	22.4 ± 0.5 ^b^	23.5 ± 0.1 ^b^	24.1 ± 0.4 ^b^	22.9 ± 0.1 ^b^
quercetin-3-rutinoside	73.3 ± 2.7 ^a^	28.9 ± 0.3 ^bcd^	27.5 ± 0.3 ^cd^	32.0 ± 0.8 ^bc^	33.5 ± 0.1 ^b^	29.6 ± 0.1 ^bcd^	29.5 ± 0.3 ^bcd^	25.2 ± 0.4 ^d^	27.9 ± 0.5 ^cd^
quercetin-3-galactoside	113.2 ± 22.2 ^a^	23.8 ± 0.1 ^b^	16.8 ± 0.0 ^b^	24.4 ± 0.1 ^b^	27.8 ± 0.4 ^b^	28.3 ± 0.0 ^b^	26.3 ± 0.6 ^b^	25.1 ± 0.5 ^b^	27.0 ± 0.5 ^b^
quercetin-3-glucoside	112.7 ± 19.7 ^a^	24.5 ± 0.1 ^b^	20.5 ± 0.2 ^b^	26.2 ± 0.8 ^b^	28.0 ± 0.5 ^b^	29.9 ± 0.3 ^b^	31.7 ± 7.8 ^b^	28.3 ± 2.3 ^b^	26.8 ± 0.1 ^b^
Anthocyanins									
cyanidin-3-galactoside	223.6 ± 33.8 ^a^	48.6 ± 0.0 ^b^	49.5 ± 0.1 ^b^	48.4 ± 0.1 ^b^	68.5 ± 0.1 ^b^	23.7 ± 0.9 ^b^	17.3 ± 0.6 ^b^	16.8 ± 0.2 ^b^	15.2 ± 0.0 ^b^
cyanidin-3-glucoside	50.5 ± 10.1 ^a^	15.8 ± 0.2 ^b^	15.0 ± 0.3 ^b^	15.7 ± 0.1 ^b^	19.7 ± 0.4 ^b^	3.0 ± 0.8 ^b^	0.8 ± 0.1 ^b^	n.d.	n.d.
unknown 1	12.1 ± 0.3 ^a^	4.2 ± 0.2 ^bc^	4.4 ± 0.1 ^bc^	5.1 ± 0.0 ^b^	5.5 ± 0.1 ^b^	5.3 ± 1.1 ^b^	4.6 ± 0.2 ^b^	2.7 ± 0.1 ^d^	3.2 ± 0.1 ^cd^
cyanidin-3-arabinoside	222.8 ± 20.8 ^a^	40.4 ± 0.3 ^bc^	36.3 ± 0.1 ^bc^	37.8 ± 0.1 ^bc^	53.6 ± 0.1 ^b^	10.3 ± 2.7 ^c^	3.4 ± 0.1 ^c^	0.7 ± 0.1 ^c^	0.5 ± 0.0 ^c^
cyanidin-3-xyloside	73.9 ± 25.3 ^a^	11.7 ± 1.3 ^b^	11.5 ± 2.2 ^b^	10.3 ± 0.4 ^b^	13.5 ± 0.2 ^b^	n.d.	n.d.	n.d.	n.d.
Flavan-3-ols									
(-)-epicatechin	159.0 ± 19.4 ^a^	61.4 ± 1.1 ^b^	65.8 ± 1.9 ^b^	65.6 ± 0.0 ^b^	62.7 ± 1.1 ^b^	66.6 ± 10.8 ^b^	65.1 ± 11.3 ^b^	64.8 ± 0.3 ^b^	58.4 ± 3.5 ^b^

The extractions were performed in two parallel samples, each measured twice (*n* = 4). The digestion process was performed in five parallel samples, which were combined and analyzed twice (*n* = 2). Different letters in the same row indicate statistically different amounts according to the post hoc Tukey test (*p* < 0.05). n.d. not detected, fw = fresh weight.

**Table 2:** Inhibition of α-amylase and α-glucosidase by phenolic compounds after intestinal digestion without or with added cellulose.

	α-Amylase	α-Glucosidase
Digestion	*IC*_50_	*IC*_50_
	μmol	μmol
Intestinal	0.041 ± 0.002 ^a^	0.020 ± 0.001 ^a^
intestinal cellulose 1	0.006 ± 0.002 ^d^	0.016 ± 0.001 ^a^
intestinal cellulose 2	0.026 ± 0.001 ^b^	0.023 ± 0.005 ^a^
intestinal cellulose 3	0.015 ± 0.001 ^c^	0.017 ± 0.001 ^a^

Samples were analyzed twice (*n* = 2). Different letters in the same column indicate statistically different values according to the post hoc Tukey test (*p* < 0.05).

**Table 3:** Solubility (%) and swelling degree (%) of cellulose in simulated fluids.

Fluid	Solubility	Swelling Degree
simulated salivary fluid (SSF)	3.6 ± 2.1	359.2 ± 40.0
simulated gastric fluid (SGF)	2.7 ± 0.7	314.0 ± 6.5
simulated intestinal fluid (SIF)	1.6 ± 0.1	386.2 ± 16.6

Samples were prepared in two parallel (*n* = 2).

**Table 4:** The correlation between phenolic subgroups (μmol L^−1^) after the digestion with cellulose and the enzyme inhibition (*IC*_50_ in μmol).

	R^2^	
Phenolic Subgroup	α-Amylase	α-Glucosidase
phenolic acids	0.925	0.477
flavonols	0.796	0.484
anthocyanins	0.691	0.276
flavan-3-ols	0.279	0.103
total	0.909	0.466

## Data Availability

The original contributions presented in this study are included in the article/Supplementary Material. Further inquiries can be directed to the corresponding author.

## Supplementary Materials

The following supporting information can be downloaded at: https://www.mdpi.com/article/10.3390/molecules31132250/s1, Table S1: Correlation between the amounts of cellulose (g) and phenolic compounds (mg kg^−1^ fw) after gastric digestion (Pearson correlation coefficient); Table S2: Correlation between the inhibition of α-amylase and α-glucosidase (*IC*_50_ in μmol), amounts of cellulose (g), and phenolic compounds (mg kg^−1^ fw) after intestinal digestion (Pearson correlation coefficient).
