# Supplementary material for: The Effects of Cellulose on α-Amylase and α-Glucosidase Inhibition by Aronia melanocarpa Phenolic Compounds After Simulated Digestion
Source: Molecules. 2026 Jun 26;31(13):2250. doi: 10.3390/molecules31132250 (PMC13362568; doi:10.3390/molecules31132250)
Supplement: Supplementary file 1 [file molecules-31-02250-s001.zip › molecules-4373313-supplementary.pdf]

Table S1. Correlation between the amounts of cellulose (g) and phenolic compounds (mg kg^{-1} fw) after gastric digestion (Pearson correlation coefficient).

	cellulose	phenolic acids	flavonols	anthocyanins	flavan-3-ols	total
cellulose	1					
phenolic acids	0.800	1				
flavonols	0.679	0.121	1			
anthocyanins	0.761	0.567	0.689	1		
flavan-3-ols	0.205	0.384	-0.270	-0.408	1	
total	0.904	0.966	0.356	0.747	0.223	1

Digestion was conducted without or with added three different levels of cellulose.

Table S2. Correlation between the inhibition of α-amylase and α-glucosidase (IC_{50} in mol), amounts of cellulose (g), and phenolic compounds (mg kg^{-1} fw) after intestinal digestion (Pearson correlation coefficient).

	-amylase	glucosidase	cellulose	phenolic acids	flavonols	anthocyanins	flavan-3-ols	total
-amylase	1							
glucosidase	0.551	1						
cellulose	-0.521	-0.001	1					
phenolic acids	0.493	-0.094	-0.957	1				
flavonols	-0.102	-0.332	-0.471	0.516	1			
anthocyanins	0.658	0.013	-0.910	0.948	0.353	1		
flavan-3-ols	0.210	0.214	-0.424	0.349	0.562	0.423	1	
total	0.495	-0.080	-0.958	0.995	0.561	0.953	0.434	1

Digestion was conducted without or with added three different levels of cellulose.
